# MAP/ERK Signaling in Developing Cognitive and Emotional Function and Its Effect on Pathological and Neurodegenerative Processes

**DOI:** 10.3390/ijms21124471

**Published:** 2020-06-23

**Authors:** Héctor Albert-Gascó, Francisco Ros-Bernal, Esther Castillo-Gómez, Francisco E. Olucha-Bordonau

**Affiliations:** 1 UK Dementia Research Institute, Department of Clinical Neurosciences, University of Cambridge, Hills Road, Cambridge CB2 0AH, UK; ha437@medschl.cam.ac.uk; 2 U.P Medicina, Facultad de Ciencias de la Salud, Universitat Jaume I, Avda. de Vicent Sos Baynat s/n, 12071 Castelló de la Plana, Spain; fros@uji.es (F.R.-B.); escastil@uji.es (E.C.-G.); 3 Spanish National Network for Research in Mental Health, Centro de Investigación Biomédica en Red de Salud Mental (CIBERSAM), Planta 0, 28029 Madrid, Spain

**Keywords:** learning, memory, hippocampus, septum, long term potentiation, long term depression, receptor, synapse

## Abstract

The signaling pathway of the microtubule-associated protein kinase or extracellular regulated kinase (MAPK/ERK) is a common mechanism of extracellular information transduction from extracellular stimuli to the intracellular space. The transduction of information leads to changes in the ongoing metabolic pathways and the modification of gene expression patterns. In the central nervous system, ERK is expressed ubiquitously, both temporally and spatially. As for the temporal ubiquity, this signaling system participates in three key moments: (i) Embryonic development; (ii) the early postnatal period; and iii) adulthood. During embryonic development, the system is partly responsible for the patterning of segmentation in the encephalic vesicle through the FGF8-ERK pathway. In addition, during this period, ERK directs neurogenesis migration and the final fate of neural progenitors. During the early postnatal period, ERK participates in the maturation process of dendritic trees and synaptogenesis. During adulthood, ERK participates in social and emotional behavior and memory processes, including long-term potentiation. Alterations in mechanisms related to ERK are associated with different pathological outcomes. Genetic alterations in any component of the ERK pathway result in pathologies associated with neural crest derivatives and mental dysfunctions associated with autism spectrum disorders. The MAP-ERK pathway is a key element of the neuroinflammatory pathway triggered by glial cells during the development of neurodegenerative diseases, such as Parkinson’s and Alzheimer’s disease, Huntington’s disease, and amyotrophic lateral sclerosis, as well as prionic diseases. The process triggered by MAPK/ERK activation depends on the stage of development (mature or senescence), the type of cellular element in which the pathway is activated, and the anatomic neural structure. However, extensive gaps exist with regards to the targets of the phosphorylated ERK in many of these processes.

## 1. Introduction

The mitogen-activated protein kinase (MAPK) superfamily is a cell-mediated signaling pathway which connects inputs to the cell, with modifications in gene expressions leading to changes in the cellular phenotype. To mediate its function, the MAPK signaling pathway is composed of three kinds of kinases, with each of them consisting of multiple members, i.e., the extracellular regulated kinases (ERKs), the Jun amino-terminal kinases/stress-activated kinases (JNKs/SAPKs), and the p38 kinase.

The MAPK/ERK signaling pathway in the central nervous system works at two different stages: throughout development and in adulthood. Throughout development, the pathway is sensitive to driving signals from the environment, promoting differentiation migration guidance to the final fate and developing the final cellular phenotype [1]. Once configured, the MAPK/ERK pathway subserves mechanisms of the transmission of cellular signal and plastic changes, including those related to learning and memory processes. The shift between these two stages has not yet been analyzed in detail.

Throughout development, the type of system that may induce the MAPK signaling pathway and the target of activated ERK may differ in organ and cellular type, so the configuration of the cellular mechanisms that may induce ERK activation and the putative targets of the activated ERK may be specific for a particular organ in a particular moment and give the system a particular rule. In addition, during pathological conditions, the ERK pathway may subserve mechanisms leading to the misfunction of cellular mechanisms associated with this disease.

Therefore, keeping in mind the double role of ERK throughout development and in adulthood, this review is centered on the mechanisms of ERK activation/inhibition which modulates and drives the central core of social and emotional behaviors, i.e., the amygdala and amygdala-related areas. We will analyze the specific function of ERK in the configuration of the emotional-related anatomical areas and in the processing of emotional and social information. Finally, we will consider the pathologies associated with ERK alterations.

## 2. Neural Signaling Mechanisms of the MAPK/ERK Pathway

In the nervous system, as a general rule, the MAP-ERK pathway can be activated by two types of extracellular signals which operate through two types of receptors, i.e., G-protein coupled receptors (GCPR) and the receptor tyrosine kinase (RTK). Additionally, as a general rule, signaling systems operating through RTK are triggered by neurotrophins responsible for neurodevelopment, while neurotransmitters trigger GPCR which convey plastic and operative machineries in adult neural cells. Table 1 summarizes the main actions and functions mediated by MAP/ERK activation after the binding of ligands to either RTK or GPCR receptors.

**Figure 1:**
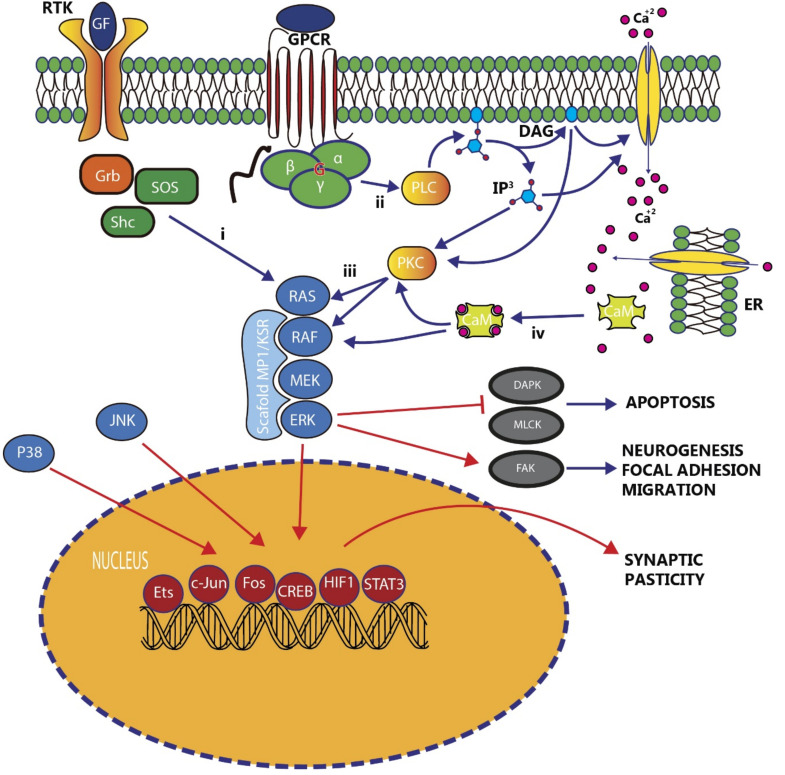
Schematic showing the main metabolic pathways in which MAPK/ERK signaling is involved. The ERK pathway can be activated by two main receptors, i.e., receptor tyrosine kinase (RTK) (i) and G-protein coupled receptor (GPCR) (ii). In addition, cytosolic mechanisms can also activate the pathway through protein kinase C (PKC) (iii) or calcium-calmodulin (CaM) (iv). The activation of the pathway can be directed by scaffolding proteins that hold together the components of the pathway. Once ERK is phosphorylated, cytoplasmic or nuclear changes may be induced, activating sets of genes responsible for neural differentiation, migration, the final fate, and plastic changes. Cytoplasmic p-ERK can promote processes such as apoptosis and neurogenesis.

Each MAPK is the end point of a module of three consecutive kinases that act as a cascade. The first step is the phosphorylation of an MAPK kinase kinase (MKKK), followed by the phosphorylation of an MEK kinase (MEK;MKK), which in turn activates an MAPK, such as ERKs, JNK, or p38 kinases (Figure 1). It is well-accepted that the MAPK pathway can be activated in four well-characterized manners: (i) Receptor tyrosine kinases (RTK), which phosphorylate Ras (MKKK), MEK (MKK), and ERK (MAPK); (ii) G-protein coupled receptors (GPCRs), which can be both Ras-dependent and Ras-independent; (iii) protein kinase C (PKC), which can be both Ras-dependent and Ras-independent and can also result from receptor activation; and (iv) Ca2+-mediated Ras activation through calmodulin (Figure 1). The attachment of molecular cascade enzymes to scaffolding proteins guides the sequences of phosphorylation and configures MAPK modules. Scaffolds of ERK approach two or all three members of the pathway (i.e., Ras, MEK, and ERK), in order to facilitate and accelerate MAPK phosphorylation, although restricting signal spreading. Specifically, the central core of the scaffolding module for the ERK pathway is formed of the scaffolding proteins kinase suppressor of Ras1 (KSR) and MEK partner 1 (MP1) [2].

There are two isoforms of ERK in this pathway: The 44kDa ERK1 and the 42 kDa ERK2. These two isoforms share up to an 84% sequence identity of the amino acid backbone. There has been a lot of controversy on whether there is functional compensation between both isoforms. ERK1 knockout mutants do not show relevant impairment in emotional and cognitive tests, including fear conditioning and passive avoidance [3]. Additionally, knocking ERK1 did not impair long term potentiation (LTP) in hippocampal slices [3]. These results lead us to consider that ERK2 is able to compensate for ERK1 function, at least in emotional-related functions. However, knocking the ERK2 gene resulted in the lethality of offspring, thus pointing out that ERK2 has its own specific function that cannot be compensated for by ERK1 overexpression [4]. Nonetheless, the main cause for lethality in ERK2-deficient mice was the implantation process. ERK2 ko mice could be rescued by the transgenic expression of ERK2, resulting in tetraploid aggregation, and these mutants were viable, although smaller than control littermates [5].

A deep analysis of transgenic mice reported several additional specific features for each isoform. Therefore, knocking ERK1 results in no changes in the hippocampal and amygdala plasticity, probably due to compensatory mechanisms, but results in the enhancement of LTP facilitation in the nucleus accumbens, which results in the enhancement of striatal-dependent long-term memory [38]. Furthermore, knocking ERK1 results in the enhancement of ERK2 expression, which drives an increase in the sensitivity to the rewarding properties of morphine [38]. On the other hand, the conditional specific inhibition of ERK2 induces a dramatic reduction of the cortical thickness as a result of the impaired proliferation of neural progenitors, which leads to profound associative learning deficits [39]. However, double mutant ko for ERK1 and conditional ko for ERK2 results in an enhanced reduction of the cortical thickness over the reduction induced by knocking ERK2 alone [40]. Therefore, it follows that the two isoforms of ERK maintain particular properties in particular cell types and in different circumstances, each one can partially compensate for the deficiency of the other isoform.

Once activated, ERK may remain in the cytoplasm and activate cytosolic pathways or translocate to the nucleus to activate different genes. Additionally, throughout this process, p-ERK may dimerize, which adds new aspects to be considered. Most of the research on targets of activated ERK has been studied in tumorigenic cell lines, but the findings of such a fundamental process in cell biology are still relevant to neuronal systems.

Several proteins that anchor ERK to the cytoplasmic compartment have been described. One of these elements is MAP kinase phosphatase 3 (MKP3). The overexpression of a catalytic inactive form of MKP3 results in anchoring pERK to the cytoplasm b, which does not alter its capacity to phosphorylate cytoplasmic substrates, but is unable to activate Elk-1-dependent gene transcription, initiating DNA replication in response to growth factors [41]. Furthermore, the unphosphorylated ERK is able to interact with actin filaments through the IQ Motif Containing GTPase Activating Protein 1 (IQGAP1) scaffolding protein [42]. This association has been observed to be relevant in the plastic changes occurring in dendritic spines and shafts in the hippocampus during contextual fear conditioning [43].

Cytoplasmic targets of pERK are involved in apoptosis and neurogenesis. It has been found that the growth factor induction of ERK activation during development maintains proliferation differentiation and the survival of neural cells, while the withdrawal of growth factors activates p38 and JNK pathways that lead to apoptosis mechanisms [31,32,44]. These effects could be mediated through mutual interactions with myosin light-chain kinase (MLCK) or death-associated protein kinase 1 (DAPK), which also participate in activating apoptotic mechanisms in anoxic conditions [45,46,47]. Another putative target of pERK is focal adhesion kinases that could be involved in neurogenesis and the migration of oligodendrocytes [48].

pERK has a small size and is able to move into the nucleus through diffusion along nuclear pores, and this nuclear translocation is a highly dynamic process [49]. Nuclear translocation has only been studied in non-neural cells, but it is expected that the process may use the same mechanisms. In addition to the MEK phosphorylation site, ERK can be phosphorylated by a particular nuclear translocation signal by casein kinase 2 (CK2); this second phosphorylation allows binding to importin-7, which acts as a carrier through the nuclear pore [50].

Once in the nucleus, pERK is able to activate different transcription factors, which leads to the expression of different genes. The types of gene that can be activated by pERK will depend on the type of cell and the moment of development. In addition, the activation of a particular gene may depend on the level of pERK reached in the nucleus, for example, transient ERK activation results in c-fos transcription, but its product cannot be accumulated and become unstable. Only sustained ERK activation is able to phosphorylate c-fos, which reciprocally maintains ERK activation and can accumulate and drive additional signaling [51]. Several immediate early genes (IEGs), such as c-Fos, Arc, CREB, or EGR1, are associated with plastic changes related to learning and memory processes [52]. Other ERK-mediated gene activators, such as E26 transformation-specific transcription factor1 (Ets1), are associated with morphogenesis and the final fates of derivatives of the mesencephalic neural crest [53].

Given the fact that many IEGs may be targeted by activated ERK, it raises the question of which of these IEGs may be activated in a particular moment and place. This question has not been fully addressed by the literature, but several aspects can be considered. First, the promotor region of most of these IEGs contains several which are controlled by different kinases, including ERK. Therefore, the expression of a particular IEG may depend on the combined activity of multiple signaling pathways. As an example, ERK activation is required for CREB transcription, but additional factors are necessary to trigger relevant CREB transcription [54]. Therefore, these combinations may be a particular feature of a particular cell type in a given moment.

Additionally, in the particular case of the central nervous system, it needs to be considered that the topographic occurrence of events is highly relevant for cellular activity. Neurons are highly polarized cells which have been shown to locally translate proteins at dendrites in a stimuli-dependent manner independently from the somatic input [55]. Local translation has also been shown to cause broad structural plasticity, as well as acute plasticity changes. Signals received within specific networks (e.g., CA3 to CA1 at the hippocampus) reinforce specific synapses and promote IEG expression on those synapses and not on others of the same anatomical area. The ability to promote synaptic plasticity also depends on structural changes in the synapses and the traffic of their components to the particular location that needs to be modified. The receptors are one of the main components and long-term potentiation depends on the traffic of AMPA receptors to the synapses. Different levels of ERK activity have been proven to induce different components of the AMPA receptors in the active synapses [54]. 

Therefore, ERK activation is a central pathway that may drive its action at a local level by activating the local translation of mRNA, promote the trafficking of synaptic components to facilitate plasticity, or induce IEG activity in the nucleus. The availability of the transcription factors that can be activated by ERK depends on the cell type, brain region, and life stage.

**Figure 2:**
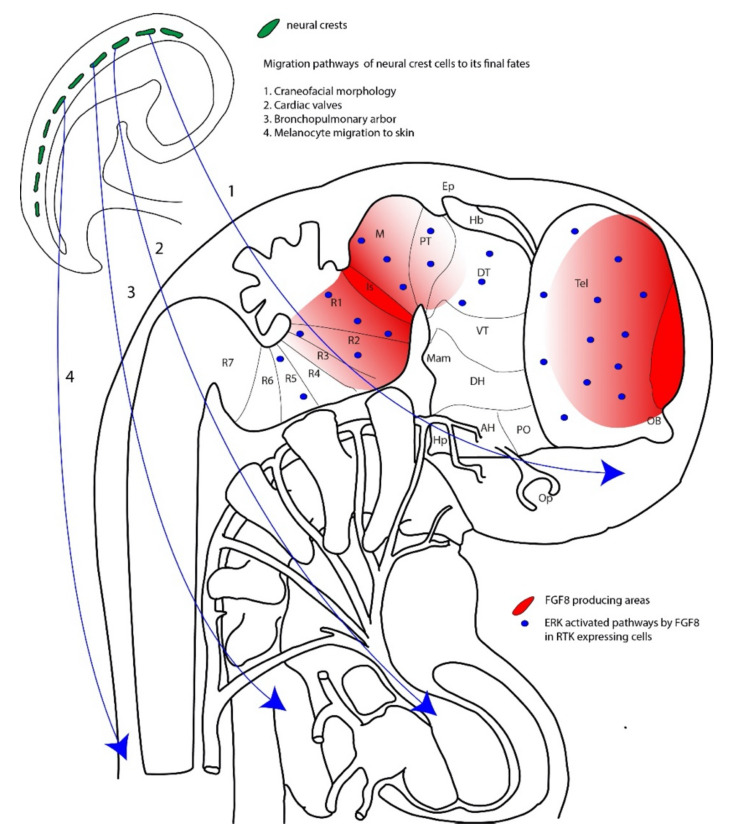
Role of MAPK/ERK signaling in embryonic development. Signals mediated by ERK promote the migration of neural crest cells to their final fates, so that disturbance of this signaling pathway may result in alterations of craniofacial morphology (1), cardiac valves (2), the bronchopulmonary system (3), and the melanocyte distribution in the skin (4). Additionally, the encephalic patterning depends, in part, on encephalic signals transmitted from the isthmus (Is) and the anterior ridge (AR), which create a gradient of FGF8 that can trigger pERK signaling in different segments of the neural axis.

There is much evidence that links ERK malfunction to neurological syndromes associated with autism spectrum disorders [56]. This evidence comes from studies of humans and animal models. In humans, altered sequences in the genes that codify for MAPK result in a constellation of changes that include cranial, lung, and heart malformations and cutaneous alterations [57]. In transgenic animal models, especially those targeting the ERK2 gene in a conditional way, an altered phenotype with disrupted emotional and social relationships is also evident [4,58] (Figure 2).

## 3. The Role of ERK in Emotional Brain Development

ERK participates in two stages of brain development that can be conceptualized as embryonic and early postnatal development. In the embryonic period, FGF8 signaling along ERK participates in proliferation, migration, and the final fate of neural cells, which results in the configuration of the main brain subdivisions [24,25,26,27,28,29,30]. In postnatal development, MAPK/ERK signaling configures the specific networks for the correct processing of emotional signals.

### 3.1. The Role of MAPK/ERK Signaling in Embryonic Development

Mutations affecting MAPK/ERK signaling pathways result in a wide range of alterations, including skin pigmentation, heart and lung alterations, skull malformations, and alterations in the configuration of the encephalic vesicle. These kinds of alterations come from the pattern of ERK expression in early development, which includes the neural crest cells whose derivatives migrate to the skull, skin (melanocytes), heart (cardiac valves), and bronchopulmonary system (tracheal and laryngeal cartilages and vegetative innervation) (Figure 2). In the central nervous system of vertebrates, FGF8 is strongly expressed in the isthmus region and anterior ridge, which are the main organizers for prosomeric and rhombomeric regions that give rise to the forebrain and midbrain rostrally and hindbrain caudally [59].

The patterning of the brain depends, to a great extent, on direct FGF8-ERK signaling. FGF8 is a morphogen in the sense that it is produced in specific regions of the developing encephalic vesicle and generates a gradient over the surrounding areas. Then, the activation of tyrosine kinase receptors (TKR) in the targeted areas generates a cascade of signaling that finally results in ERK phosphorylation, which, in turn, initiates mechanisms of cell proliferation, differentiation, and migration to the final fate. FGF8 is highly expressed in two secondary organizers, i.e., the isthmus (Is) in the boundary between the midbrain and rhomboencephalon and the anterior neural ridge (ANR), which is the edge of the anterior part of the neural plate located between the roof and the floor plates primordia during and after neural tube closure. On the other hand, ERK is highly phosphorylated at both sides of the isthmus and in the area just caudal to the rostral tip of the telencephalon [59]. Therefore, FGF8-ERK signaling is crucial for the development of rhombomeric and mesencephalic patterning on one side and pallial and subpallial morphogenesis and patterning on the other side.

There are up to three tyrosine kinase receptors that can bind FGF8, i.e., FGFR1-3 [60]. The FGF8 signal can reach its targets by two mechanisms, i.e., simple diffusion and the sink function of the targeted cells regulated by receptor-mediated endocytosis [61]. The transduction of the FGF8-receptor can be carried out by three signaling pathways: PI3Kinase, PLC-gamma, and Ras/MAPK [62]. In the Ras-MAPK pathway, the pattern of ERK phosphorylation correlates with FGF8 expression domains [63]. Throughout the morphogenesis process, the timing of the morphogen synthesis and delivery and the receptor availability are relevant. In addition to the position, the temporal pattern is also relevant. To this end, apoptosis plays a relevant role in determining the number of FGF8-producing cells. Supporting this view, the overexpression of FGF8 from the anterior neural ridge has been found in mutant mice for apoptotic genes (apaf1). This overexpression resulting from a maintained population of FGF8-producing cells results in impaired gene expression in the ventral forebrain [64].

The FGF8-ERK signaling system directs the processes of the regional configuration of telencephalic areas, and once developed, the general patterning and morphogenesis of the main forebrain and midbrain divisions. FGF8 signaling on radial glial cells of the developing thalamus induces ERK1/2 activation in the radial fibers which guide the proliferation and migration of neural progenitors from the thalamic mantle layer to the final fate, in order to generate the thalamic map. Reduced FGF8 production results in a decreased number of thalamic cells and deficient thalamocortical innervation, and this process is parallel to a reduction in ERK phosphorylation in the developing thalamus [65].

**Figure 3:**
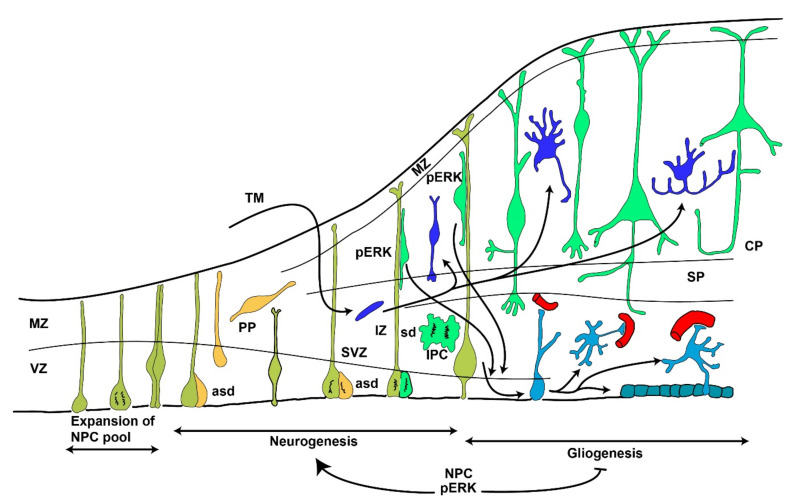
Scheme showing the development of the cerebral cortex and the role that MAPK/ERK signaling may have at different stages. Three main stages can be defined. The first stage is characterized by continuous divisions resulting in the expansion of the neural progenitor cell (NPC) pool. Starting at E11 until E17, the neurogenesis process is driven by ERK phosphorylation in the NPC, which suppresses gliogenesis. During neurogenesis, two kinds of divisions can be seen, i.e., asymmetric divisions (asd) and symmetric divisions (sd). Neuronal progenitors resulting from asd can derive intermediate progenitor cells (IPC) which divide in sd and cells resulting from these divisions migrate along the shafts of radial glia to the final fate. Inhibitory neurons in the cerebral cortex arise from tangential migration (TM) from the ganglionic eminences. Immature neurons produce signaling trophic factors mediated by MAPK/ERK signaling which induce gliogenesis, resulting in the generation of the different types of astrocytes. Throughout brain development, the cortex becomes layered from an initial period only composed of the ventricular zone (VZ) and the marginal zone. Then, newly born neurons are located in an intermediate layer that originates from the preplate (PP), which differentiates into the subventricular zone (SVZ), intermediate zone (IZ), and subplate (SP). Then, the area containing the neuronal bodies of neurons makes the cortical plate. Modified from [67].

Another aspect that is disrupted when the MAPK/ERK signaling pathway is altered is the organization of the cerebral cortex. The genesis of the mammalian cerebral cortex consists of at least two basic processes (Figure 3): On one side, the cells that originate in situ by the proliferation and differentiation of neural cells from the ventricular zone, and on the other side, inhibitory neurons that originate from tangential migration from the subpallial ganglionic eminences [66,67]. MAPK/ERK signaling is one of the main mechanisms involved in the in situ generation and differentiation of neural cells required to configure the cerebral cortex. Alterations in MAPK/ERK signaling do not produce prominent changes of the cortical structure, but result in alterations in the number of neurons and the neuron/astrocytic rate, thus leading to thicker or thinner cortices [39,68].

The cerebral cortex originates from neural progenitor cells (NPCs) within the ventricular zone (VZ). In the first phase, the NPCs of the VZ produce neurons and later on, glial cells. During the first neurogenesis phase, the self-renewing multipotent NPCs divide in two ways, i.e., symmetric and asymmetric divisions. Symmetric divisions produce two cells that differentiate towards neurons. Asymmetric divisions produce a cell-renewing progenitor and a daughter cell, which may differentiate towards a neuron or stay as an intermediate progenitor cell (IPC). In turn, the IPCs may undergo another asymmetric or symmetric division to continue the process [69,70].

Newly born neurons secrete stimuli that induce gliogenesis from undifferentiated NPCs in later phases of embryogenesis [71]. During the early phases, signals transmitted through ERK in progenitor cells promote neurogenesis and repress gliogenesis [71]. Manipulations in the MAPK/ERK signaling pathway disrupt the normal size cortex [72,73]. It has been demonstrated that inhibition of the upstream kinase for ERK and MEK blocks neurogenesis and means that NPCs remain as undifferentiated cells in the subventricular zone [73]. These effects can also be produced by inactivating scaffolding proteins that engage the tyrosine kinase receptor in the MAPK/ERK system [74]. Additionally, the conditional inactivation of ERK2 during neurogenesis results in the generation of fewer neurons and many more astrocytes [39].

Control of the cell cycle is one of the key elements in controlling the final number of cortical neurons. Several experiments have shed light on this aspect using murine models of 16p11.2 deletion. This model mimics the human syndrome of this deletion in humans that accounts for the 1% of cases of autism spectrum disorders [75]. The 16p11.2 interval contains the encoding region for ERK1, but, paradoxically, this manipulation results in an enhanced expression of ERK2 [76]. This overexpression drives altered levels of the downstream effectors of the signaling pathway cyclin D1 and p27Kip, which control the cell cycle during mid neurogenesis. Cells resulting from the increased proliferation of neuronal progenitors leave the cycle and enter the differentiation process, which results in the early depletion of progenitor pools [76]. Therefore, transgenic mice carrying the 16p11.2 deletion (16p11.2del) display smaller brains, a thinner cortex, and autistic features [76]. These alterations can be prevented by the ip application of ERK inhibitors (RB1 and RB3 peptides) to 16p11.2del mutant pregnant dams between gestation days E10 and E14 [68].

In addition to the in situ development of the cortex, MAPK/ERK signaling also contributes to the generation and final fate of cortical interneurons by regulating the generation of progenitors in the medial ganglionic eminence (MGE) and caudal ganglionic eminence (CGE) [77]. In these areas, neural progenitors give rise to undifferentiated cells that migrate tangentially to reach the cortex [66,78]. The specification for an interneuronal fate is regulated, among other factors, by members of the Dlx gene family and compound mutations in Dlx1/Dlx2 display a severe reduction in cortical GABAergic cells at birth [79].

### 3.2. The Role of MAPK/ERK Signaling in Late Embryonic and Early Postnatal Development

Once configured, the main components involved in emotional and social processing need to maturate to produce the final architecture subserving those functions. The maturation process consists of several biochemical and morphological changes that result in the extension of extrinsic and intrinsic connections, developing the dendritic trees and spines and apoptotic mechanisms to produce the final number of neurons in a given center. These processes are highly regulated by several pathways of intracellular signals in which ERK is also involved. In fact, a dynamic regulation of MAPK phosphorylation pathways throughout postnatal development has been found in the hippocampus [80]. Specifically, three peaks of increased activities of p38MAPK at postnatal ages of 4 (PN4), 10 (PN10), and 21 (PN21) were found. ERK continuously increased the total p-ER from PN1 to PN60, but considered alone, the phosphorylation of ERK2 started to display a significant increase from PN14. It is noteworthy that this time window corresponds to an increase in synaptogenesis [81]. Considering the same postnatal ages, no variations were observed for the JNK1-3 pathway [80].

Alterations of the normal maturation process may come from a stressful condition, such as maternal separation. In this paradigm, a reduction of brain-derived neurotrophic factor (BDNF) synthesis which results in a reduction of MAPK/ERK signaling through the TrkB pathway has been described. As a final result of this manipulation, a reduction of spines in hippocampal neurons has been found [28].

The relevance of ERK phosphorylation in early postnatal development and its consequences for adult behavior were also tested by an intraperitoneal injection of the MEK inhibitor SL_327_. In these cases, a blockade of the ERK pathway at PN6 resulted in an increase of forebrain apoptosis and long-lasting deleterious effects of normal behavior in adulthood, including social recognition, impaired memory, and reduced LTP. No such deficits were observed when the blockade was performed at or over PN14 [82]. Neural survival and apoptosis are also mechanisms regulated by insulin and IGF signaling through ERK pathway [23].

One important aspect of the final configuration of neuronal typology is the dendritic branching. BTBR T+tf/J (BTBR) is an inbred strain of mice that has been extensively used as a model of autism as these animals display several autistic-like behavioral features, including deficits in social communications and interaction and repetitive stereotyped behaviors [83]. In these animals, an abnormal growth of the dendritic tree has been found, which could be associated with dysregulated MAPK/ERK signaling [83]. When analyzing the structure of the pyramidal CA1 neurons, BTBR neonatal mice displayed longer apical and basal dendrites with a higher branching complexity. Biochemical analysis during this period also showed increased ERK phosphorylation [83].

A role of ERK phosphorylation has also been postulated in the synaptogenesis occurring during postnatal development. A high period of synaptogenesis around PN15 has been found [81]. During this period, interfering 5HT signaling through 5HT1A-receptors impairs normal synaptogenesis through a mechanism involving ERK and PKC pathways [84].

### 3.3. The Role of MAPK/ERK Signaling in Adult Emotional and Memory Systems

The ubiquity of ERK expression in the CNS rises the possibility of participation of this pathway in multiple cognitive processes (Table 2). The activation of the pathway in particular areas may drive the cellular function towards different directions. In the case of the amygdala it could be considered as the central core for emotional processing being social behavior and fear the two main studied emotions. MAPK/ERK signaling pathways provide mechanisms for both types of emotions and have been seen to regulate them. In addition, most of these processes are context-dependent and the contextual aspects of the emotional processing are directed from the entorhino-hippocampal system that also shares basic mechanisms of memory processing through ERK pathways.

#### 3.3.1. MAPK/ERK Signaling in Spatial Memory

The first experiment which studied ERK kinases and LTP together was carried out in the hippocampus, one of the most relevant regions which integrates and stores memory after being encoded at other areas. Many have divided the hippocampus, based on its role in information processing, into the dorsal and ventral hippocampus. While the dorsal hippocampus obtained inputs from the medial septal area, known as the entorhinal cortex, the ventral hippocampus is the only one receiving projections from the amygdala. Inputs to both these subareas point out a primordial role in spatial memory of the dorsal division of the hippocampus, while the ventral hippocampus processes social memory [96,97].

The extensive work on “place cells” mostly focused on the CA1-CA3 fields of the hippocampus is a good and illustrative example of how critical the pivotal role of the ERK pathway in LTP generation in the dorsal hippocampus and spatial memory formation is [85,86]. To test spatial memory, many different spatial tasks have been used, from two-trial alternation tests on T-maze, Y-maze, or radial maze, to others such as the Morris water maze. Regardless of the task in place, several experimenters have highlighted the specific activation of the ERK pathway, showing ERK phosphorylation on pyramidal neurons on the dorsal CA1-CA3 fields of the hippocampus. Conversely, animals have shown no ERK activation on pyramidal neurons at the ventral hippocampus during these tasks [85].

This distinct activation of the MAPK/ERK pathway between the dorsal and ventral hippocampus further supports the idea of them having distinct roles in memory formation and processing. These different roles come from multiple organizational differences. The main input to the dorsal and ventral hippocampus comes from two functionally different areas of the entorhinal cortex [98]. Moreover, the dorsal and ventral hippocampus receive different innervation from brainstem modulatory areas, such as the raphe nucleus [99] carrying serotonin [100] and the nucleus incertus [101,102] carrying relaxin3 [19]. Additionally, dorsal and ventral hippocampal dentate gyri display different levels of adult neurogenesis [103]. The dorsal hippocampus shows higher densities of proliferating cells and newborn granule cells. This faster maturation in the dorsal hippocampus correlates with higher levels of neural network activity in this region. In contrast, a higher production of granule cells in the ventral hippocampus is associated with chronic stress and antidepressant interventions [104]. It is well-established that all of the factors that decrease adult hippocampal neurogenesis impair spatial learning, while enhanced adult neurogenesis is related to increased spatial learning. In this sense, MAPK/ERK might act as a rheostat to modulate adult neurogenesis [105].

On the other hand, infusion into the dorsal hippocampus of specific ERK inhibitors (PD098059) impairs consolidation, but not acquisition, of spatial memory. Inhibition of the consolidation of memory then leads to an impairment of long-term memory. The inhibition of long-term memory is caused by ERK inhibition, which leads to decreased local protein synthesis rates. The reduction in local protein rates then leads to the non-reinforcement of specific synaptic systems [87]. The inhibition of other kinases, such as p38, did not impair memory formation or consolidation for long-term memory [85]. This further highlights the specific role of MAPK in memory formation and consolidation in the hippocampus. Similarly, ERK inhibition with SL327 causes an attenuation of learning in a water maze paradigm, which is not observed if injections of the inhibitor are given after the task [86]. This makes the inhibition of ERK1/2 specific to the stage of encoding and consolidating memory formation.

In the dentate gyrus (DG), BDNF-induced LTP increases the activation of BDNF receptor TrkB and ERK when comparing young and aged animals [33,106]. However, the activation of MAPK/ERK signaling pathways is not always indicative of spatial memory consolidation, as many neurotransmitters activate metabotropic receptors, such as the G coupled receptor with Gαi/o, which inhibit key neurons for spatial memory consolidation. This is the case for neurotransmitters such as GABA, which binds to the GABA_B_ receptor at DG, CA1, CA2, and CA3 fields, inhibiting pyramidal and granular neurons, respectively, but increasing the activation of ERK1/2 [107,108]. Likewise, infusions of the neuropeptide relaxin-3 (RLN3), which binds with a high affinity to RXFP3, an inhibiting GPCR, causes an increase in ERK activation on cholinergic neurons at the medial septum septohippocampal pathway [14]. Cholinergic neurons are known to promote a theta rhythm, which precedes place cell firing and as such, spatial memory formation [109].

Altogether, ERK’s role does not seem to exclusively be as a spatial memory generator, but more as a signal transduction modulator. Good examples of this are the promotion and reinforcement at place cells at CA1, inhibition through the activation of GABAergic interneurons at the hippocampus, and the inhibition of cholinergic neurons at the medial septum. This is why the ERK1/2 pathway appears to play a role as an integrator of signals which ensures the correct convergence and reinforcement of memories, being both a generator and inhibitor of such memories.

#### 3.3.2. MAPK/ERK Signaling in Social Behavior

If ERK activation plays a role in which stimuli are reinforced and which plastic changes take place in spatial memory, this is also the case in social behaviors. This memory is stored and processed in the ventral hippocampus, amygdala, hypothalamus, and bed nucleus of the stria terminalis (ST). As in the case of spatial memory, the nature of this reinforcement largely depends on the context, meaning the type of neuron, receiving stimuli, and the surrounding context which integrates these stimuli [110].

During social behavior, the MAPK/ERK pathway is mostly activated when encoding social-related memories. However, some cases exist when only testing social interaction in rodents, which does not necessarily involve memory. These experiments show a decrease in social interaction measured by olfactory exploration times after the inoculation of SL327 (an MEK1/2 inhibitor) in olfactory bulbs. The greatest impact of the MAPK/ERK pathway on social behavior has been seen in social recognition memory. Social recognition memory is the process of social behavior which allows a subject to remember an already acquainted conspecific. Protein synthesis is required when information regarding social stimuli is integrated and stored in long-term memory. The specific details of the experimental set up may lead to different outcomes. For example, social recognition memory requires two stages of protein synthesis at olfactory bulbs, medial amygdala, and the pyriform cortex when introducing juveniles as novel social stimuli [111]. In these experiments, the use of anysomycin blocked social recognition memory 20 mins before the encounter, immediately after and 6 h after. However, it had no effect 3 or 8 h after the encounter, suggesting two events of protein synthesis. The authors concluded that the first stage of protein synthesis takes place 1–2 h after the social encounter, while the second one takes place 6–7 h after the encounter. While, in the first stage, there is a clear activation of c-Fos, indicative of neuronal activity, in the areas of social stimuli processing, the second stage does not exhibit such activation [111]. How these events relate to ERK activation and whether it is more specific to the first event or the second is still not known.

Complementary studies on the medial amygdala show that only in the case of long-term (24 h) social recognition memory is protein synthesis required for the consolidation of memory [88]. Nonetheless, it is difficult to compare the experiments due to differing approaches in experimental designs (e.g., anysomycin inoculation times). Altogether, the results lead to the conclusion that ERK activation might not only be exclusively dependent on memory, but also on the nature of the tested behavior, its encoded memory, and specific stimuli that promote it.

On the other hand, ERK is activated in different variants of social behavior, such as aggressive behaviors, social anxiety, and social defeat [89,112,113]. Furthermore, the activation of the oxytocin receptor (OXTR) drives the raf-ERK at the paraventricular nucleus in lactating rat females to elicit anxiolytic behaviors [89]. Furthermore, activation with the direct inoculation of oxytocin (OT) in the paraventricular nucleus causes a decrease in anxiety in an elevated plus-maze task, which is disrupted in the presence of the MEK1/2 inhibitor (U0126, 0.5 nmol/0.5 µL) injected prior to OT inoculation [90]. Other studies using short day photoperiods to elicit aggressive behavior events in both male and female rodents also showed an increase in pERK1/2 when photoperiods were short compared to when they were long. This increase in pERK1/2 was located at the medial nucleus of the amygdala (MeA) and nucleus of the stria terminalis (ST) [112]. Finally, the isolation of rats leading to depressive phenotypes also showed a decrease in pERK at the hippocampus. Although no memory tasks were tested, it is still possible that in all of these cases, memory processes such as the stages of protein synthesis and neuronal plasticity are taking place downstream of ERK activation/deactivation. 

As for the case of spatial memory, in social behavior, ERK activation seems to stimulate neuronal plasticity reinforcement on specific synapses. The nature of this reinforcement seems to be dependent on stimuli convergence. In the case of social behavior, considering its overlap with other systems, such as anxiety, stress, and aggression, this convergence might be even more complex. For example, the oxytocin and relaxin-3 pathway lead to opposite effects on social recognition memory; while oxytocin promotes it [12], RLN3 impairs it [15]. At the same time, relaxin-3 is known to be involved in the response to stress [114]. It is especially relevant as both systems show an increase in ERK activation upon the binding of either the OXTR or RXFP3, respectively [13,20]. These data further support the role of ERK as an integrator of stimuli which helps encode memory, depending on which systems are activated.

#### 3.3.3. MAPK/ERK Signaling in Fear

There is abundant literature on the role of MAPK signaling in plasticity mechanisms related to fear conditioning. Pioneer studies showed MAPK cascade involvement in NMDA-dependent LTP [115]. Although most LTP studies have used either the performant pathway on the dentate gyrus or Schaffer’s collateral to CA1 as the anatomical background, in which high-frequency stimulation provides plastic synaptic sensitization, the thalamic input to lateral and basal amygdala is another point where these changes may take place [91]. It is noteworthy that the application of the MEK inhibitor UO126 to the incubation bath of a slice preparation disrupted LTP when high-frequency stimulation was applied to the thalamo-amygdala pathway [92]. This result correlates with intracerebroventricular (icv) injections of PD098059 (MEK inhibitor), which disrupted the consolidation of both contextual and cued fear conditioning in a dose-dependent manner. However, the short-term memory remained intact, indicating that ER is necessary for memory consolidation [116]. The role of ERK in the consolidation of fear memory was additionally assessed as the performance of auditory fear conditioning resulted in a transient, but significant, increase in ERK phosphorylation one hour after task acquisition [116]. Moreover, it has been found that in vivo high-frequency (but not low-frequency) stimulation of the thalamo-amygdala pathway is able to induce LTP in the lateral amygdala. Moreover, intra-lateral amygdala infusion of another MEK inhibitor (U0126) impaired LTP at the thalamo-amygdala synapses [117]. Therefore, ERK activation is a basic condition required to promote the plastic changes of the thalamo-amygdala synapses that may support amygdala fear learning.

ERK activation takes place in a specific manner, which relates to areas regulating the learning process. The involvement of ERK in the retrieval of fear memories has been demonstrated in the inhibitory avoidance paradigm, where an increase of pERK has been shown in areas such as the hippocampus, entorhinal, and anterior cingulate cortex [118]. Moreover, the infusion of an MEK inhibitor into the hippocampus during an inhibitory avoidance task impaired memory expression and blocked extinction during successive tests, both before and after retrieval infusion [119]. Furthermore, blocking pERK and other signaling systems impaired extinction, regardless of whether blocking was done before the first retrieval session or after it during the extinction trials [120]. ERK pathway is also required in the extinction of aversive memories mediated by opioids and other forms of plastic changes related to addiction [22,35,36]. Therefore, ERK signaling displays a role in retrieval, as well as during the extinction process.

MAPK/ERK signaling also plays a relevant role during the reconsolidation process. Throughout this process, fear memories need to be consolidated again after being activated [121]. Interfering with the process of re-consolidation just after the already consolidated memory has produced activated results in delating this memory [121]. Infusions of U0126, an MEK inhibitor, in the amygdala just after the retrieval of a cued fear memory resulted in the impairment of memory reconsolidation [93]. In the same way, the systemic administration of SL327, another MEK inhibitor, shortly after the retrieval of a trace memory, also resulted in the impairment of reconsolidation in a dose-dependent manner [94]. In this same work, the authors showed that MAPK/ERK signaling plays a relevant role in both consolidation and reconsolidation, as ERK1 ko mice displayed enhanced levels of freezing in both retrieval and re-consolidation and post-retrieval administration of the MEK inhibitor impaired the reconsolidation enhancement observed in the ERK1 ko mice [94]. Moreover, a synaptic-specific action in memory reconsolidation that occurred in the intra-amygdala infusion of U0126 just before the retrieval of one of two of the already acquired auditory cues resulted in specific impairment of the reconsolidation towards this specific cue and not the unretrieved cue [95]. In addition, the administration of U0126 before retrieval induced a reduction in the auditory-evoked field potential of the activated conditioned stimuli (CS) [95].

Consolidation and reconsolidation processes depend on de novo protein synthesis that, in part, is triggered by MAPK/ERK signaling systems [122]. However, the pathways for memory consolidation and reconsolidation may be different in the hippocampus and amygdala [34]. Only the dentate gyri display similar patterns for contextual fear conditioning after training or retrieval [123]. However, different patterns of IEG activation (such as Arc, egr-1, or c-fos) were observed after consolidation and reconsolidation downstream of ERK activation [123].

## 4. MAPK/ERK Dysfunction in Neurodegenerative Diseases

As we have previously described, MAPK signaling pathways intervene and control cellular functions, resulting in a direct function of memory and emotional processes. Therefore, alterations or modulations of these pathways can lead to different processes implicated in various human diseases. Throughout this and the next section, we will analyze the state-of-the-art of MAPK signaling pathways in human disease, with a special focus on neurodegenerative disorders (Table 3) and autism.

The role of the MAPK/ERK pathway in neurodegenerative diseases is mainly related to glial cell function and the inflammatory response. The activation of resident immune cells of the brain, glial cells (microglia and astroglia), triggers the pro-inflammatory state with the production of nitric oxide (NO), cytokines, and chemokines and the implication of inflammatory-related pathways [124,125,126]. Most of the components of these pathways are cytosolic targets of ERK, suggesting an essential function of the MAPK pathway in the production or sustaining of such a pathological hallmark, and consequently, in the noxious events that lead to the specific neurodegeneration.

### 4.1. Parkinson’s Disease

Parkinson’s disease (PD) is an age-associated disease mostly identified by an extrapyramidal alteration of movement. From a pathological point of view, PD is characterized by the selective and progressive loss of dopaminergic-melanized neurons located in caudoventral regions of the substantia nigra, reactive gliosis, and intracytoplasmic inclusions of α-synuclein known as the Lewy bodies [127]. In this sense, α-Synuclein promotes inflammation via activating p38, ERK, and JNK pathways in human microglial cells, resulting in the production of IL-1β and TNF-α. The disappearance of neurons in the substantia nigra leads to dopamine deficiency in their target areas (in the striatum and other nuclei of the basal ganglia), producing serial functional lesions and the manifestation of symptoms and clinical signs. Many genes, including 23 genes or loci linked to rare monogenic familial forms of PD with Mendelian inheritance, such as SNCA, Parkin, DJ-1, PINK 1, LRRK2, and VPS35, and over 20 common variants with small effect sizes and 12 genetic risk factors, have been associated with PD in recent years [128,129].

Leucine-rich repeat kinase 2 (LRRK2), also known as dardarin, is a 2527 amino acid (~280 kDa) protein that, in humans, is encoded by the PARK8 human gene and constituted by several functional domains, including leucine-rich repeats, an Ras-related GTPase domain, an MAP3K domain, and multiple potential protein interaction domains [130]. Several mutations in the Ras-related GTPase and MAP3K domains of LRRK2 have been associated with familial and idiopathic PD [131]. In this sense, the G2019S mutation is the most common pathogenic mutation associated with the familial form of PD, representing about 3% of cases overall (40% in some populations). The LRRK2 locus has also been associated with idiopathic PD (iPD), as an oxidative mechanism selectively increased wild-type LRRK2 kinase in both the substantia nigra from iPD patients and in two different rat models of the disease [154]. Although all MAPKs participate in neurodegeneration associated with LRRK2, ERK is the most plausible downstream mediator of mutant LRRK2 effects [130]. In this regard, it has been observed that the dysregulation of dopaminergic neurodegeneration-related genes in induced pluripotent stem cells derived from PD patients harboring a G2019S mutation could be minimized by ERK inhibitors [131]. Additionally, during the last decade, an increase in pERK in leucocytes from patients carrying the G2019S mutation [132], the presence of cytoplasmic granules of pERK in Lewy body aggregates in the substantia nigra of LRRK2 G2019S PD patients [130], and a G2019S-LRRK2-associated neurite retraction triggered by ERK-dependent mechanisms in a PD in vitro model have been described [155].

The impact of ERK in PD-associated neurodegeneration has also been analyzed using the most relevant animal models of parkinsonism, both neurotoxins 6- OHDA and 1-Methyl-4-phenyl-1, 2,3,6-tetrahydropyridine (MPTP), suggesting that ERK may contribute to the pathogenesis of neurodegeneration.

6-OHDA remains the most widely used tool to induce a selective nigrostriatal lesion in murine models and dopaminergic cell lines. In this regard, B65 6-OHDA-induced cell death depends on chronic ERK activation, and this dopaminergic death can be mostly attenuated using an MEK blocker [133]. Conversely, when the same cell line is treated which hydrogen peroxide, also inducing transient ERK activation, MEK blockers are ineffective for modifying cell death [133]. Moreover, a recent study revealed the inhibition of L-DOPA-induced dyskinesias (LID) following the counteraction of ERK in the dopamine-depleted striatum of 6-OHDA-treated mice [134].

MPTP is a compound that, having passed through the blood–brain barrier, is catabolized by astrocytes to its neurotoxic form MPP+ and causes permanent symptoms of parkinsonism selectively affecting dopaminergic neurons in the substantia nigra. The addition of MPP+ to neuroblastoma cell lines increases α-synuclein, induces the activation of ERK, and triggers cell death that can be reverted using the MEK-P inhibitor U0126 [135]. As the action of this inhibitor excludes altering α-synuclein levels, it seems that both ERK activation and α-synuclein pathways are independent [156]. In this sense, ERK is almost exclusively activated in the microglia localized in striatum and substantia nigra pars compacta of MPTP-treated mice [157]. Moreover, the administration of Galectin-1, with an anti-neuroinflammatory effect, to MPTP-treated mice resulted in microglial p38 and ERK1/2 dephosphorylation, followed by IκB/NFκB signaling pathway inhibition ameliorating the neurodegenerative process [136].

Finally, the implication of the ERK pathway in PD is beyond animal models. The substantia nigra of PD patients presents phosphorylated-ERK associated with fibrillar bundles inside coarse discrete cytoplasmic granular accumulations surrounding Lewy bodies, suggesting a potential interaction between the mitochondrial function and the MAPK/ERK signaling pathway in dopaminergic neurodegeneration [137].

### 4.2. Alzheimer’s Disease

Alzheimer’s disease (AD) is the most common form of dementia and the most prevalent neurodegenerative disease [158]. AD is a neurodegenerative disorder of an unknown etiology characterized by the progressive loss of memory and other cognitive functions that lead to dementia. The brains of AD patients have several distinctive neuropathological features: Intracellular neurofibrillary tangles (NFTs), whose main component is the abnormally phosphorylated tau protein [159]; senile plaques (SP), primarily consisting of beta-amyloid (Aβ) [160]; and neurodegeneration [161], especially relevant in the basal telencephalon, the origin of cortical and hippocampal cholinergic innervation [162,163]. Besides, the disease progresses through a reduction of synaptic proteins [164], changes in the synaptic morphology and structure [160], and neuroinflammation [165]. AD usually occurs sporadically, but approximately 5–10% of patients manifest it in a familiar way.

MAPK pathways differentially activate during AD. All three MAP-kinases are implicated in mild and severe cases (Braak stages III–VI), both ERK and JNK/SAPK are implicated in Braak stages I and II and in non-demented cases without pathology hallmarks (Braak stage 0), and either ERK alone or JNK/SAPK alone can be activated [166]. This different participation suggests that both oxidative stress (JNK/SAPK and p38) and mitotic signaling alterations (ERK) are independently able to initiate, but both are necessary to disseminate, disease pathogenesis.

Amyloid β, the principal component of amyloid plaques, constitutes the main link with ERK pathway activation. In this sense, it has been established in both in vivo and in vitro studies that chronically elevated levels of Aβ induce the dysregulation of hippocampal ERK MAPK [138,139]. Additionally, increased p-ERK was revealed in brain extracts of AD patients [140]. On the other hand, the oxidative stress induced by Aβ activates p38 MAPK and triggers the hyperphosphorylation of tau, which is the other main neuropathological hallmark in AD [167].

Interacting with both AD-associated proteins, the α7 nicotinic acetylcholine receptor (α7nAChR) binds to soluble amyloid-beta, resulting in tau phosphorylation and the formation of neurofibrillary tangles. Moreover, α7nAChR mediates the activation of p38 MAPK and ERK1/2 signaling pathways, suggesting an essential role of both α7nAChR and MAPK signaling pathways in the uptake and accumulation of β-amyloid [141].

Furthermore, during the last decade, it has been suggested that mitochondrial dysfunction is an early pathological feature of AD related to oxidative stress and Ca2+ homeostasis that triggers Aβ-induced synaptic dysfunction [168]. It has been proved that heme oxygenase-1 (HO-1) plays a role in protecting neurons against Aβ-induced oxidative stress [142]. Recent studies have demonstrated that acteoside induces HO-1 expression through Nrf2 activation. This activation depends on ERK and PI3K/Akt pathways, but not on JNK and p38MAPK pathways [169].

However, the role of ERK in AD is not clear, since an increase of total ERK, specifically within synaptosomes, is associated with a deficient memory task performance in AD transgenic mice [143]. In this sense, the activation of ERK, downstream of NMDA NR2B receptor activity, plays an interesting role in regulating memory processes [144]. Moreover, alterations in NR2B phosphorylation and MAPK/ERK signaling induce beta amyloid-associated behavioral deficits in an AD murine model [145]. Recently, it has been demonstrated that changes in synaptosome MAPK/ERK signaling following ACE2-activator administration increased signaling through the NR2B receptor, inducing significant protection against cognitive decline and decreasing the amyloid accumulation [146].

### 4.3. Amyotrophic Lateral Sclerosis and Huntington’s Disease

Amyotrophic lateral sclerosis (ALS), a term proposed by Charcot in 1874 [170], is a degenerative neurological disease that affects the pyramidal pathway along its first and second motor neurons and results in the progressive loss of bulbar and limb function. Therefore, the existence of lateral sclerosis involves the damage of projection axons of the first motor neuron and amyotrophic damage of the second motor neuron. The diagnosis of this pathology is primarily clinical [171], classically reflected in the criteria of El Escorial of 1998 [172]. Moreover, in 2008, electromyographic criteria were defined as a diagnostic tool for second motor neuron injury, despite the absence of semiological findings pathologically (Awaji criteria) [173]. Most ALS cases are sporadic; however, around 10% of cases may be familial due to mutations in genes, including those for Cu/Zn superoxide dismutase 1 (SOD1), dynactin, TAR DNA binding protein 43 (TDP-43), and chromosome 9 open reading frame 72 (C9orf72) [174]. Although the latest research suggests that p38 and JNK MAPK play a determinant role in ALS [175], ERK pathway alteration is also related, since SOD1(G93A) transgenic mice present a dysregulation in axonal transport associated with the down-regulation of ERK correlating with the up-regulation of JNK and caspase-8 [147].

Huntington’s disease (HD) is one of nine autosomal dominant neurological diseases caused by an expansion mutation of CAG triplets encoding polyglutamine (polyQ) sequences in N-terminal domains. It affects 3–7 cases per 100,000 of the Western Europe population, and its symptoms include motor disorders (chorea and stiffness, among others), cognitive disorders (subcortical dementia), and psychological disorders (such as irritability and depression), which end with the death of patients. While the wild-type huntingtin (Htt) protein modulates intracellular vesicular trafficking and neuronal development, mutant Htt, with an elongated polyQ domain, generates toxic N-terminal fragments after undergoing proteolytic processing [176].

Mutant Htt presents kinase downstream ERK deficiency involved in transcriptional dysregulation and by triggering striatal degeneration, it also decreases the response to cortico-striatal BDNF signaling and downregulates ERK-dependent glutamate transporter expression, increasing cells susceptible to glutamatergic excitotoxicity [148,149].

### 4.4. Prion Diseases

A prion is the altered form of a 23-kDa constitutive protein (PrP in mammals) that has lost its normal function, but has acquired the property of transforming the standard form into a pathological form. This protein has a regular conformation called PrPc, encoded by a gene (PRNP) localized to human chromosome 20. In prion pathologies or prionopathies, an altered isoform originating as a result of the incomplete proteolysis of PrPc, called PrPsc, tends to form amyloid aggregates in the form of plaques in the brain. Prionopathies are disorders of the conformation of proteins, which manifest themselves as spongiform encephalopathy in animals, such as scrapie, and as neurodegenerative diseases in humans. The accumulation of PrPsc causes the involvement of the gray matter with neuronal death, gliosis, and spongiform changes. Activated microglia is a classic hallmark of neuroinflammation associated with prions, as these cells phagocytize and eliminate amyloid plaques [177,178]. As a part of the neuroinflammatory scenario, activated microglial cells regulate MAPK signaling pathways [179].

Scrapie-infected hamster’s brains present an up-regulation of both pJNK and pERK [180]. ERK is neuroprotective following prion infection, since the inhibition of phospho-ERK triggered the death of scrapie-infected cells. Even more, membrane-resident PrP proteins trigger phospho-ERK activation [150]. After prion infection, there is an increased level of the phospho-ERK complex, but this is also related to a decrease in MEK complex activation, suggesting a divergent action of some phosphatases on ERK1/2 upon chronic prion infection [151].

## 5. MAPK/ERK Signaling and Autism Spectrum Disorders

As we have previously described, MAPK signaling pathways intervene and control cellular functions resulting in a direct function of memory and emotional processes. Therefore, alterations or modulations of these pathways can lead to different processes implicated in various human diseases. Throughout this and the next section, we will analyze the state-of-the-art of MAPK signaling pathways in human disease, with a special focus on autism and neurodegenerative disorders.

From the classical definition proposed by Kanner in 1943 [181], and by Asperger in 1944 [182], to the present, the concept of autism has undergone many variations. Although the nuclear symptoms of autism have remained unchanged over time, leading experts in the field have considered the associated symptoms with different criteria. Autism as a specific and symptoms-delimited disease does not exist, since it has no specific biological markers, and lacks pathophysiology or a clear etiology that explains it. Therefore, autism comprises a constellation of symptoms derived from a dysfunction of the central nervous system, with significant variation in the degree of intensity (autism spectrum disorders, ASD) [183].

Recent studies support the critical role of MAPK/ERK signaling in metabolism, where this pathway intervenes in the regulation of cell metabolism, since alterations in MAPK/ERK signaling are associated with metabolic syndrome [184]. In this regard, ASD pathway network analysis has demonstrated the existence of eight functional metabolism pathways and one metabolic disease pathway, constituting nearly 22.5% of the network [56]. Moreover, it has been well-established that ASD patients have a high risk of suffering from or developing metabolic problems as comorbidities, even during the prenatal stage [185,186]. In this respect, the “calcium-PKC-Ras-Raf-MAPK/ERK” pathway emerges as a novel relation with ASD, as this pathway is implicated in heart diseases and metabolic problems that are already related with or lead to an increased familial risk for ASD [56]. Moreover, there is an enhancement of MAPK/ERK signaling proteins in ASD patients [187].

On the other hand, MAPK/ERK is also implicated in the stabilization of dendritic spines, since it participates in encoding adhesion molecules and scaffolding proteins [188,189]. In this regard, it has been suggested that impaired cognition is due to an excitatory and inhibitory synaptic disbalance that could also possibly be implicated in ASD [190,191]. In this sense, BTBR mice show positive p-Erk1/2 immunolabeling levels in the prefrontal cortex that negatively correlate with cognitive function [152].

Finally, recent studies suggest the existence of a critical window connecting alterations in MAPK/ERK activity and autism, since the blockade of MAPK/ERK signaling at postnatal day 6 in rats induces autistic behavioral phenotypes that are not present at day 14 [82,153].

## 6. Conclusions

The MAPK/ERK signaling pathway in the central nervous system participates at two different stages: Throughout development and in adulthood. During these periods, different transcription factors can be activated by MAPK/ERK, depending on the type of cell and the moment in life.

In embryonic and early postnatal phases, signals transmitted through MAPK/ERK in progenitor cells elicit neurogenesis and repress gliogenesis [71]. In this sense, there is a narrow window between PN6 and PN14 when the blockade of the MAPK/ERK pathway induces an increase of forebrain apoptosis and long-lasting deleterious effects, including social recognition, impaired memory, and reduced LTP in adulthood [82].

ERK participates in different neuronal processes, such as spatial memory, social behavior, and fear conditioning. While ERK elicits a signal transduction modulator role in spatial memory, activated ERK stimulates neuronal plasticity reinforcement on specific synapses in social behavior. On the other hand, MAPK/ERK signaling systems, which follow different pathways in the hippocampus and amygdala, trigger crucial de novo protein synthesis for consolidation and reconsolidation processes [122].

It was previously believed that in terms of the simplistic term-dependent ERK function, while the early activation of ERK was thought to be typically neuroprotective, delayed and chronic ERK activation were understood to promote neuronal cell death [192]. However, recent evidence indicates that ERK activation alone may not be predictive of the subsequent cellular response.

In addition to the chronic activation of ERK, its localization within the cell seems to play a crucial role in initiating neurodegenerative processes [193].

However, it is difficult to directly associate the MAPK/ERK signaling pathway to a specific neurodegenerative disease as this pathway is part of a more extensive network of other intricate signaling pathways. On top of that, ERK also interacts with more than 70 different cytoplasmic and nuclear substrates, modulating many fundamental cellular processes, such as cortical neurogenesis, proliferation, and excitation, which may have a cumulative effect on cognitive and behavioral impairments. 

Many pathologies of the central nervous system have been associated with disruption of the MAPK/ERK signaling pathway, in agreement with the ubiquity of this enzyme in neurons and glial elements. Some of these syndromes are directly related to ERK gain/loss of function and in other cases, ERK implication is indirectly a consequence of the wide spectrum of MAPK/ERK signaling pathways participating in central nervous system homeostasis.

## Figures and Tables

**Table 1:** Physiological function of the activation of the microtubule-associated protein kinase or extracellular regulated kinase (MAPK/ERK) pathway via G-protein coupled receptors (GCPR) or receptor tyrosine kinase (RTK) receptors.

**Ligand**	**Receptor/Type**	**Process**	**Reference**
GABA	GABAB-R/GPCR	Long-term inhibition of synaptic transmission	[6,7,8]
		Association with trafficking	
		Developing brain	
Glu	m-Glu/GPCR	Fine control over glutamate activity	[8,9]
		Synaptic plasticity	
		Developing brain	
CRH	CRH-R/GPCR	Response to stress	[10,11]
OXT	OXTR/GPCR	Promotes social recognition memory	[12,13]
		Anxiolytic	
RLN3	RXFP3/GPCR	Inhibits social recognition memory, and spatial short-term memory	[14,15,16,17,18,19,20,21]
		Stress response	
		Anxiolytic	
		Food intake	
Cannabinoid	CB1-GPCR	Addiction	[22]
INS/IGF1	INSR-IGF1R /RTK	Mitogenesis, glucose uptake, and metabolism	[23]
		Neuronal survival, neuronal and glia development, and synaptic plasticity	
		Reproductive endocrinology	
FGF8	FGFR1-3 TrkA-B/RTK	Axonal growth and regeneration	[24,25,26,27,28,29,30]
		Neural and neural crest differentiation, migration, and final fate	
NGF	Trk-A/RTK	Neuronal survival	[31,32]
BDNF	Trk-B/RTK	Neuronal and glia developmentand synaptic plasticity	[33]
		Memory	[34]
		Addiction	[35]
		Addiction-extinction	[36]
EGF	EGFR/RTK	Development and maintenance	[37]
		Appetite suppression and neuroendocrine alterations	
		Acute and chronic pathological processes	

**Table 2:** ERK1/2 activation in behavior.

Behavior	ERK (+) Activation (−) Inhibition	Reference
Spatial memory	(+) LTP generation, spatial learning (**−**) impairs effects	[85,86]
	(**−**) PD098059 infusion inhibits long-term memory formation	[87]
Social recognition memory	(+) promotes social recognition memory	[88]
Elevated plus maze	(+) activation at PVN causes an anxiolytic effect (**−**) eliminates the anxiolytic effect	[89,90]
Fear conditioning	(**−**) disrupts LTP at the amygdalo-thalamic pathway	[91,92]
Consolidation of fear memory	(**−**) infusion of U0126 prior to the retrieval of memory impair reconsolidation	[93,94,95]

**Table 3:** Participation of Erk in neural and neurodegenerative diseases.

	**Parkinson’s disease**	**References**
LRRK2	p-ERK present in Lewy bodies	[130,131,132]
6-OHDA model	6-OHDA elicits sustained ERK phosphorylation related to LID	[133,134]
MPTP model	ERK phosphorylation is implicated in neuroinflammation	[135,136]
PD patients	ERK phosphorylated deposits close to Lewy bodies	[137]
	**Alzheimer’s disease**	
AD patients	Aβ dysregulates hippocampal ERK	[138,139,140]
SH-SY5Y cells	α7nACh induce tau phosphorylation and neurofibrillary tangle formation after binding to soluble Aβ	[141]
PC12 cells	HO1 protects against Aβ-induced oxidative stress	[142]
Transgenic mice	ERK-signaling induces Aβ-associated behavioral deficits	[143,144,145,146]
	**ALS and HD**	
SOD1 transgenic mice	ERK is down regulated, which induces a dysregulation in axonal transport	[147]
Mutant Htt model	ERK deficiency triggers striatal degeneration and increases glutamate susceptibility	[148,149]
	**Prion diseases**	
Prion infected mice	ERK is neuroprotective following prion infection	[150,151]
	**Autism**	
BTBR mice	p-ERK immunolabeling negatively correlates with cognitive function in the prefrontal cortex	[152]
ERK 1 /2 KO mice	Critical window between 6^th^ and 14^th^ postnatal day when an ERK blockade promotes autistic behaviors	[82,153]

Aβ: beta amyloid; AD: Alzheimer’s disease; ALS: amyotrophic lateral sclerosis; HD: Huntington’s Disease; Htt: huntingtin; LID: Levodopa-induced dyskinesia; PD: Parkinson´s disease.

## Abbreviations

5HT	Serotonin
AD	Alzheimer’s disease
ALS	Amyotrophic lateral sclerosis
asd	Asymmetric division
ASD	Autism spectrum disorders,
Aβ	Amyloid β
BDNF	Brain-derived nerve factor
CA1–3	Cornu ammonis fields 1–3
CaM	Calmodulin
CGE	Caudal ganglionic eminence
CP	Cortical plate
CS	Conditioned stimuli
CREB	c-AMP response element binding protein
DAG	Diacyl glycerol
DAPK	Death-associated protein kinase 1
DG	Dentate gyrus
Dlx	Distal-less homeobox
ER	Endoplasmic reticulum
ERK	Extracellular regulated kinase
Ets	E26 transformation-specific transcription factor
FAK	Focal adhesion kinase
FGF8	Fibroblast growth factor 8
GABA	Gamma amino butyric acid
GF	Growth factor
GPCR	G-coupled protein receptors
Grb	Growth factor receptor bond protein
HD	Huntington’s disease
HIF1	Hypoxia-inducible factor 1 transcription factor
HO-1	Heme oxygenase-1
Htt	Huntingtin
IEG	Immediate early gene
IP3	Inositol 3-phosphate
IPC	Intermediate progenitor cell
iPD	Idiopatic Parkinson´s disease
IQGAP1	IQ Motif Containing GTPase Activating Protein 1
IZ	Intermediate zone
IκB	Inhibitor of κB
JNK	c-jun N-terminal kinase
JNKs/SAPK	Jun amino-terminal kinases/stress-activated kinases
KSR	Kinase suppressor of Ras1
KSR	Kinase suppressor of Ras1
LID	L-DOPA-induced dyskinesias
LRRK2	Leucine-rich repeat kinase 2
LTD	Long-term depression
LTP	Long-term potentiation
MAPK	Microtubule-associated protein kinase
MeA	Medial Amygdala
MEK	MAP-ERK kinase
MGE	Medial ganglionic eminence
MKP3	MAP kinase phosphatase 3
MLCK	Myosin light-chain kinase
MP1	MEK partner 1
MPTP	1-methyl-4-phenyl-1,2,3,6-tetrahydropyridine
MZ	Marginal zone
NFT	Neurofibrillary tangles
NFκB	Nuclear factor kappa-light-chain-enhancer of activated B cells
NPC	Neural progenitor cell
OT	Oxytocin
OXTR	Oxytocin receptor
PD	Parkinson’s disease
PKC	Protein kinase C
PN1–60	Postnatal day 1–60
PP	Preplate
PRNP	Gene encoding prion protein
PrP	Prion protein
RAF	Rapidly Accelerated Fibrosarcoma
Ras	Rat sarcoma small GTPases protein
RAS	Rat sarcoma small GTPases gene
RLN3	Relaxin 3
RTK	Receptor tyrosine kinase
SBZ	Subventricular zone
sd	Symmetric division
Shc	Src homolog and collagen homolog
SOD1	Cu/Zn superoxide dismutase
SOS	Son of sevenless homolog a guanine exchange factor
SP	Subplate
ST	Bed nucleus of the stria terminalis
STAT3	Signal transducer and activator of transcription 3
TM	Tangential migration from ganglionic eminences
TrkB	Tyrosine kinase receptor B
VZ	Ventricular zone
α7nAChR	α7 nicotinic acetylcholine receptor
